# Clinical and neurophysiological characterization of muscular weakness in severe COVID-19

**DOI:** 10.1007/s10072-021-05110-8

**Published:** 2021-03-23

**Authors:** Francesco Bax, Christian Lettieri, Alessandro Marini, Gaia Pellitteri, Andrea Surcinelli, Mariarosaria Valente, Riccardo Budai, Vincenzo Patruno, Gian Luigi Gigli

**Affiliations:** 1 Department of Neurosciences, Udine University Hospital, Udine, Italy; 2 Department of Medicine (DAME), University of Udine, Udine, Italy; 3 Pneumology Unit, Udine University Hospital, Udine, Italy

**Keywords:** Intensive Care Unit-Acquired Weakness (ICU-AW), Critical illness polyneuropathy (CIP), Critical illness myopathy (CIM), Neuromuscular disease, Neurophysiology, COVID-19

## Abstract

**Objective:**

To report clinical and electroneuromyographic (ENMG) characteristics of patients affected by severe COVID-19 infection, evaluated for muscular weakness.

**Materials and methods:**

ENMGs performed for evaluation of diffuse weakness in patients who could not be discharged from semi-intensive care COVID unit because of difficulties in ventilation weaning were reviewed. Patients with severe COVID-19 infection who had undergone endotracheal intubation and able to co-operate were considered. ENMG protocol was focused on neurophysiological items that excluded or confirmed critical illness polyneuropathy (CIP), myopathy (CIM), or polyneuromyopathy (CIPM). Standardized clinical evaluation was performed using Medical Research Council (MRC) sum score.

**Results:**

Eight patients were included in the study. All presented known risk factors for intensive care unit-acquired weakness (ICU-AW), and none of them had history of underlying neuromuscular disorders. ENMG findings were normal in two patients, while only two patients had an altered MRC sum score (< 48). Neuromuscular involvement was diagnosed in 6/8 patients (75%): 2 had CIP, 1 had possible CIM, 1 had CIPM, while 1 patient, with clinically evident weakness but equivocal ENMG findings, was classified as ICU-AW. Finally, 1 patient was diagnosed with acute demyelinating neuropathy. Patients with neuromuscular involvement were those with longer intubation duration and higher levels of IL-6 at admission.

**Conclusion:**

Neuromuscular complications are frequent in severe COVID-19 and cannot be excluded by MRC sum scores above 48. Standardized ENMG is helpful in guiding diagnosis when clinical evaluation is not reliable or possible. Elevated IL-6 at admission may be a predictor biomarker of ICU-AW in COVID-19.

**Supplementary Information:**

The online version contains supplementary material available at 10.1007/s10072-021-05110-8.

## Introduction

During the COVID-19 pandemic, cases of neurological complications in patients with severe SARS-CoV-2 infection—and thus with prolonged stay in the intensive care unit (ICU)—have been reported. Agitation, corticospinal tract involvement, and dysexecutive syndrome are the most frequent ones. These neurological manifestations seem to be more evident after sedation and neuromuscular blocking agent withdrawal [1].

Out of the pandemic, the neurological complications due to prolonged stay in ICU are well characterized and ICU-acquired weakness (ICU-AW) is one of the most frequent and insidious. Risk factors for ICU-AW are numerous and include female sex, sepsis, multiorgan system failure, severe inflammatory response syndrome (SIRS), prolonged sedation, long duration of mechanical ventilation, immobility, hyperglycemia, glucocorticoids, and neuromuscular blocking agents [2]. According to clinical and neurophysiological findings, ICU-AW is classified in three subcategories [3]: critical illness polyneuropathy (CIP), critical illness myopathy (CIM), and, when they overlap, critical illness neuromyopathy (CIPM). Prolonged neuromuscular blockade, a reversible condition due to non-depolarizing neuromuscular blockers, must be considered in the differential diagnosis.

In our intensive care and semi-intensive care COVID units, alongside with agitation and confusion, muscular weakness was observed after extubation, requiring neurological evaluation in most cases. We report the clinical and electroneuromyographic (ENMG) characteristics of patients with severe COVID-19 infection complaining of diffuse weakness.

## Methods

In this study, we review the clinical and ENMG examinations performed at the end of the first pandemic curve for neurological evaluation in patients still present in the semi intensive care COVID unit of the Udine University Hospital. Indication to the examinations was the diffuse weakness complained by the patients or observed by the medical staff. Patients with severe confirmed COVID-19 infection who had required acute laryngeal intubation and subsequently extubated, who were still admitted due to difficulties in non-invasive ventilation weaning, were included in the study. Only the examinations performed in those patients who were able to co-operate and mentally capable to express a verbal witnessed informed consent to the neurophysiological evaluation were considered for the present study. Age at admission, sex, BMI, duration of mechanical ventilation, and Charlson comorbidity index (CCI) [4] were collected. Pharmacological therapy was reviewed, with particular regard on glucocorticoid use. Standard laboratory data were obtained, including complete blood count at admission, blood glucose, liver enzymes levels, C-reactive protein, procalcitonin, and IL-6 at admission. Neurological evaluation was focused on strength assessment, and MRC (Medical Research Council) sum score was administrated. Scores below 48/60 were considered altered [5]. ENMG was performed by using Dantec™ Keypoint® G4 workstation (Synopo®) according to the following standardized protocol: (1) nerve conduction studies (NCS) including three motor nerves (ulnar, peroneal and tibial nerves-either side), five sensory nerves (superficial radial, III digit median, V digit ulnar—either side—and both sural nerves), F-responses of ulnar and tibial nerves, and repetitive nerve stimulation of ulnar nerve in order to exclude neuromuscular junction disorder and (2) electromyographic study of tibialis anterior and vastus lateralis muscles including qualitative analysis of spontaneous activity at rest and quantitative analysis of interference pattern [6] (IP) according to a standardized protocol (i.e. number of turns, amplitude, number of short segments, envelope). This simplified ENMG exam protocol was conceived for ICU studies in order to focus on neurophysiological items which permit to confirm or exclude CIP and CIM diagnosis. Diagnosis of CIP or CIM was made when either Bolton [7] or Lacomis [8] criteria were fulfilled. A diagnosis of unspecified ICU-AW was adopted when either clinical or neurophysiological evaluations were equivocal. Possible signs of acute demyelinating polyneuropathy were evaluated according to the Rajabally [9] criteria. All the ENMG findings were reviewed by two experienced neurophysiologists (C.L. and R.B.).

## Results

Among 40 patients admitted in the ICU for COVID-19 infection who had required endotracheal intubation, an ENMG study was requested for a total of 9 patients, still admitted for difficulties in weaning of non-invasive ventilation and complaining of diffuse weakness. One patient was excluded from the study because she was unable to cooperate. Detailed demographic, clinical, and laboratory findings of the 8 patients included in the study are summarized in Table 1. For all of them, the past medical history was negative for previous neurological disorders or any other diseases capable of causing underlying neuromuscular complications.

**Table 1:** Demographics and selected laboratory data of the study population

Patient ID	Age	Sex	BMI	Duration of intubation (in days)	CCI	C-RP (g/dL)	IL-6 at admission (pg/mL)	Pct
1	53	M	18.5	13	1	39.0	39	0.09
2	70	M	24.2	7	3	100.2	8	2.52
3	59	M	30.1	21	1	188.7	53.2	0.65
4	40	M	36.3	16	1	56.3	303	0.17
5	48	M	20.8	15	0	42.2	102	0.24
6	56	M	25.7	16	1	278.1	32	0.46
7	60	M	32.3	12	2	86.8	10	0.39
8	53	M	30.6	14	0	246.8	529.6	6

*Pct* procalcitonin, *IL-6* interleukin-6, *C-RP* C-reactive protein, *CCI* Charlson Comorbidity Index, *BMI* body mass index

Because of difficulty in maintaining spontaneous breathing, all patients had undergone endotracheal intubation and subsequently tracheostomy before complete weaning from invasive ventilation. The mean time of intubation was 14.2 days (range 7–21).

Pharmacological therapy was similar in all patients and followed our hospital COVID-19 therapeutic protocol, including antiretroviral drugs (either lopinavir-ritonavir or darunavir-cobicistat) and chloroquine/hydroxy-chloroquine. All patients except one (ID #4) were treated with steroids. Five patients received tocilizumab, in one of them associated to remdesivir, upon prescription of the infectivologist. Six patients had concomitant bacterial or fungal supra-infections that were treated with empiric or targeted antimicrobial therapy accordingly.

**Fig. 1:**
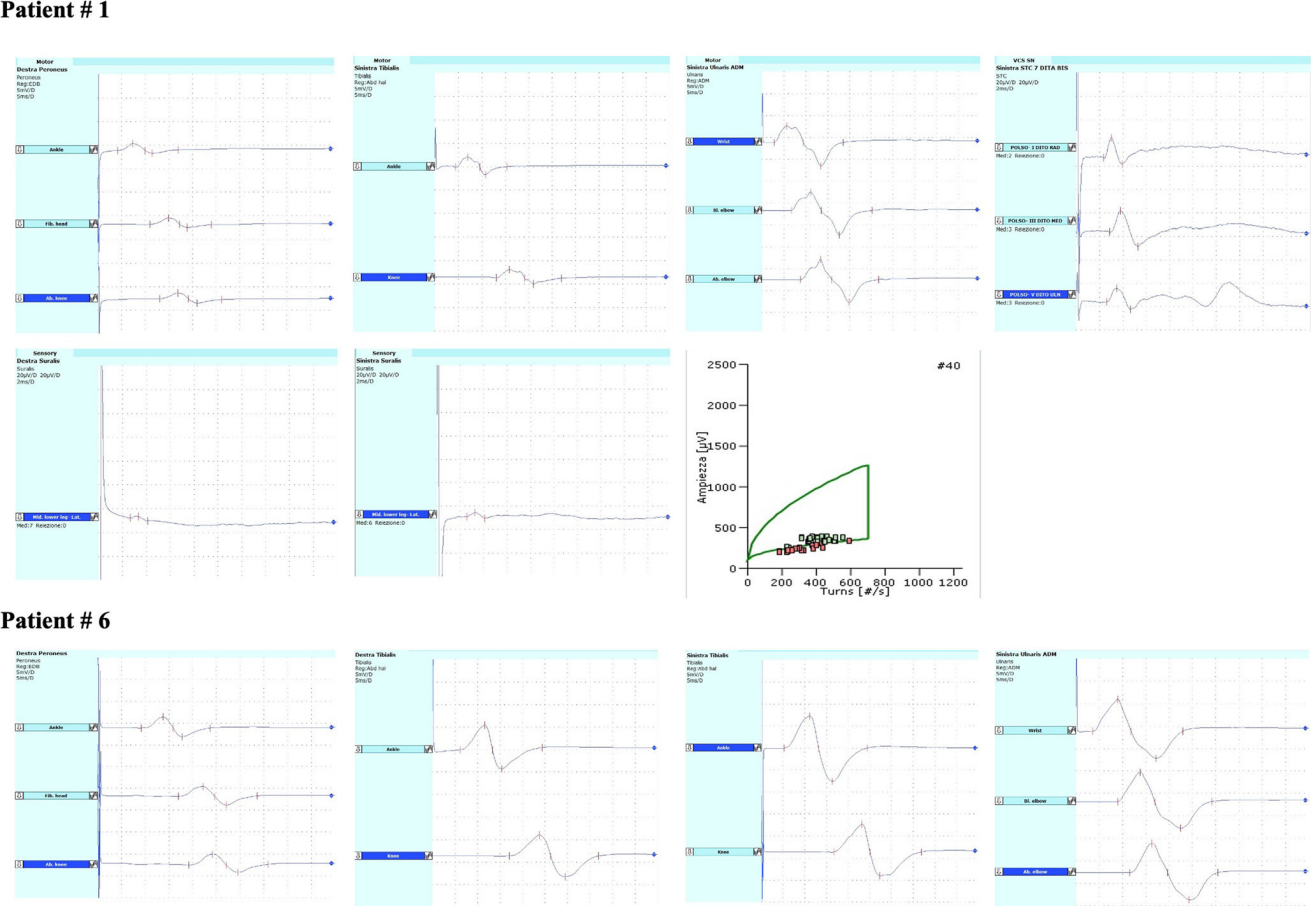
Selected NCS and turn amplitude analysis. Amplitude is measured peak-to-peak. Motor NCS: gain = 5 mV/D, LFF = 2 Hz, HFF = 10 kHz; sensory NCS: gain =20 μV/D, LFF = 20 Hz, HFF = 2 kHz. A, amplitude; DL, distal latency; CV, conduction velocity. Values abnormal according to our laboratory reference normative values are marked in bold. *Patient #1* affected by CIPM. Nerve conduction studies and turns/amplitude analysis are reported. Motor NCS of right peroneus (A = 2.1 mV, DL = 4.19 m, CV = 41.9m/s), left tibialis (A = 3.8 mV, DL = 4.4 ms, CV = 44.9 m/s), and left ulnar nerves (A = 9.2 mV, DL = 2.8 ms, CV = 55.7 m/s). Antidromic sensory NCS of both sural nerves (left: A = 4.3 μV, DL = 2.56 ms, CV = 54.7 m/s; right: A = 2.2 μV, DL = 2.62 ms, CV = 45.8 m/s) and left radial (A = 19.6 μV, DL = 2.33 ms, CV = 51.5 m/s), median (A = 22.8 μV, DL = 2.85 ms, CV = 49.1 m/s), and ulnar (A = 14.2 μV, DL = 2.6 ms, CV = 48.1 m/s) nerves are reported. Turns amplitude analysis of left tibialis anterior is shown in the last box. *Patient #6* affected by AIDP. Motor NCS of right peroneus (A = 4.6 mV, DL = 8.74 ms, CV = 38.1 m/s), right tibialis (A = 9.7 mV, DL = 6.0 ms, CV = 38.9 m/s), left tibialis (A = 14.9 mV, DL = 5.49 ms, CV = 39.3 m/s), and left ulnar (A = 13.2mV, DL = 3.37 ms, CV = 47.0 m/s) nerves

Repetitive nerve stimulation study was normal in all patients, thus excluding neuromuscular junction disorders. ENMG findings were normal in two patients who showed an MRC sum score above 48. Four patients could be diagnosed as presenting one of the subcategories of ICU-AW. In particular, two of them had CIP, one possible CIM and one CIPM (Fig. 1); among these four cases, only one patient had MRC sum score below than 48/60. One more patient with a MRC sum score of 45/60 (ID#8), although fulfilling the criteria for ICU-AW [3], presented equivocal ENMG findings. In fact, ENMG examination revealed a right peroneal neuropathy due to a possible compression/entrapment with axonal loss. For this reason, the patient was classified as ICU-AW without specifying the subcategory according to Bolton’s [7] and Lacomis [8] criteria. Patient # 7 had normal findings except for ENMG outcomes of radial and ulnar neuropathy due to a previous elbow fracture.

Finally, one patient presented ENMG findings suggestive of demyelination. At the time of clinical evaluation, this patient showed a moderate proximal and distal limb weakness, with diffuse areflexia, but without sensory symptoms. ICU physicians, as well as physical therapists, described in the same patient a flaccid tetraparesis immediately after ventilation weaning, which subsequently improved with time. The patient underwent lumbar puncture with cerebrospinal fluid analysis that showed only mild protein elevation (530 mg/L) while bacterial, viral, and fungal rapid nucleic acid test were negative. SARS-CoV-2 RNA in cerebrospinal fluid was absent. The ENMG findings, alongside with the clinical history, raised a strong suspicion of a concomitant acute inflammatory demyelinating polyneuropathy (AIDP) according to Rajabally [9] criteria (Fig. 1).

Detailed neurophysiological results are available as supplementary materials.

## Discussion

In our study population, CIP, CIM, and CIPM were the most frequently encountered neurological disorders based on clinical or neurophysiological evaluation (4/6; 66%). At the moment, we are not able to ascribe neuromuscular findings to COVID-19 infection, since all patients presented other known risk factors for ICU-AW. We can only note that none of them had previous history of any disease capable of causing underlying neuromuscular disorders, deeming unlikely that the ENMG findings would be already present before the infection.

Long duration of ICU stay, multiorgan system failure, SIRS, glucocorticoids administration, prolonged sedation, and prolonged endotracheal intubation are well-recognized risk factors for the development of neuromuscular complications, independently from the etiology causing ICU admission [2]. In our study population, most of these risk factors were present, as discussed in the results section and as shown in Table 1.

It is worth noticing that the development of neurological disorders seems to be independent from the burden of comorbidities in our series. In fact, the only two unaffected patients were just those with higher CCI scores. Our data suggest, instead, a possible association with the duration of intubation and with increased IL-6 levels at admission. In fact, although six patients presented superimposed infection during the hospital stay, the increased levels of IL-6 at admission should be regarded as marker of severity of COVID-19 infection. Moreover, as already demonstrated by Witteveen and colleagues [10], elevated IL-6 at admission is one of the risk-predictor biomarkers for developing ICU-AW, independently from sepsis. Thus, elevated IL-6 may be the sign of a vigorous immune and inflammatory response which could represent the link between severity of COVID-19 infection and ICU-AW development. Notably, the two patients with normal MRC sum score and normal ENMG findings were also those with the shortest duration of intubation and the lowest level of IL-6 at admission. These observations need to be confirmed in larger series.

On the other side, neuromuscular complications cannot be excluded in patients with MRC sum scores above 48, as shown in Table 2. In fact, only 2 out of 8 patients had a score below 48, confirming the need of a neurophysiological evaluation, since the clinical assessment of a suspected ICU-AW may not be reliable in the ICU setting [11]. It is known that neuromuscular alterations (either clinically or neurophysiologically evident) can be detected in patients admitted to the ICU for sepsis or multiorgan system failure, but who do not fulfill the ICU-AW diagnostic criteria [3, 12]. However, the relevance of these changes and how to categorize them is still debated, since the diagnostic accuracy, validity, and clinical applicability of ICU-AW criteria have not been formally investigated.

**Table 2:** Comparison between MRS sum score results and final diagnosis after ENMG examination

Patient ID	MRC sum score	Final diagnosis
1	53	CIPM
2	60	Normal findings
3	54	CIM (probable)
4	54	CIP
5	46	CIP
6	51	AIDP
7	57	Normal findings*
8	45	ICU-AW**

*ICU-AW* intensive care unit-acquired weakness

*Except for ENMG outcomes of a radial and ulnar neuropathy due to a previous elbow fracture

**ENMG findings were consistent with an outcome of an entrapment peroneal neuropathy. See text for more details

We believe that our findings should prompt diagnostic attention for peripheral nervous system disorders among patients with severe COVID-19 infection: indeed, such patients are less likely to receive a complete clinical and/or neurophysiological evaluation, due to their isolation and consequently to the technical difficulties in performing instrumental examinations. At our best knowledge, we are aware of only two cases of CIM recently reported [13, 14]. Early recognition of ICU-AW spectrum may be of crucial importance, considering that this entity is known to be associated with increased mortality and, usually, with a long incomplete recovery. Moreover, several therapeutic and physical therapy adjustments could be able to reduce mortality and clinical and neurophysiological impairment as well as the recovery time. These adjustments include aggressive sepsis treatment, aggressive glucose lowering therapy, avoidance of high-protein nutrition, enteral feeding, and early mobilization [15]. In addition to this, isolated entrapment neuropathy is not uncommon in patients in the ICU, due to loss of subcutaneous fat and/or focal compression. This may be the case of patient #8. Prevention in these cases is obtained with a proper positioning of the limb to avoid compression.

Finally, case #6 deserves special consideration. During ICU stay, this patient received intravenous amiodarone, a drug known to cause—albeit rarely—a demyelinating neuropathy. However, amiodarone-associated neuropathy develops only in 6% of patients, usually appearing after a mean time interval of 5 to 12 months since first drug administration [16]. For these reasons, we consider post-COVID AIDP to be a more reasonable diagnosis. This hypothesis is also supported by the recent description of AIDP in COVID-19 patients [17–19]. Considering the improving temporal course, this patient did not receive any immunomodulation therapy.

We are aware that our sample size is limited; however, we think it can be indicative, because of the standardized clinical and neurophysiological protocol adopted, as well as the homogeneity of the population considered.

## Conclusions

Our case series shows that neuromuscular involvement is a frequent complication in patients with severe COVID-19 infection (6/8, 75%) and that, as a consequence of the isolation measures, it may be difficult to diagnose. Neuromuscular complications are not excluded by MRC sum scores above 48 and should be actively searched. Neurophysiological evaluation is more sensitive when clinical evaluation with MRC sum score is not reliable or possible and, in this setting, it may be helpful to detect neuromuscular involvement in order to adopt any possible therapeutic measure able to reduce the recovery time and improve patients’ clinical outcome.

In case of generalized weakness during COVID-19 infection, ENMG should be performed at least when patients present with severe weakness or with unusual features such as weakness asymmetry and global areflexia.

Further studies are needed to confirm that elevated IL-6 at admission is a risk-predictor biomarker for ICU-AW developing in COVID-19, in order to foster prevention of this complication. Until then, we believe that ENMG study—when available and accessible—may still be a valuable tool to aid differential diagnosis and clinical characterization of diffuse weakness in these patients.

## Supplementary information

ESM 1 (DOCX 29 kb)

## Data Availability

The data that supports the findings of this study are available in the supplementary material of this article.
