# Supplementary material for: Clinical and neurophysiological characterization of muscular weakness in severe COVID-19
Source: Neurol Sci. 2021 Mar 23;42(6):2173–8. doi: 10.1007/s10072-021-05110-8 (PMC7985745; doi:10.1007/s10072-021-05110-8)
Supplement: Supplementary file 1 — (DOCX 29 kb) [file 10072_2021_5110_MOESM1_ESM.docx]

**Table S1: Motor conduction studies.** Ampl=amplitude (mV); DL= distal latency (ms); CV= conduction velocity (ms); FW = F-wave latency (ms); n.a.=not applicable. Values abnormal according to our laboratory reference normative values are marked in bold

| **Patient**  **ID #** | **Ulnar nerve** | **Peroneal nerve** | **Tibial nerve** |
| --- | --- | --- | --- |
| **1** | Ampl 9.2  DL 2.8  CV 55.7  FW 30.8 | Ampl **2.1**  DL 4.19  CV **41.9** | Ampl **3.8**  DL 4.4  CV 44.9  FW 56.6 |
| **2** | Ampl 11.4  DL 3.36  CV 48.3  FW 27.2 | Ampl 6.1  DL 4.27  CV 45.1 | Ampl 16.9  DL 4.09  CV 40.7  FW 55.2 |
| **3** | Ampl **6.0**  DL 2.92  CV **45.4**  FW **35.3** | Ampl **1.1**  DL **11.2**  CV 46.7 | Ampl **1.91**  DL 5.56  CV **34.5**  FW n.a. |
| **4** | Ampl 10.0  DL 3.16  CV 51.2  FW 33.4 | Ampl **3.4**  DL 5.31  CV **38.8** | Ampl **6.7**  DL 5.35  CV **38.8**  FW **64.4** |
| **5** | Ampl 8.2  DL 2.13  CV 58.0  FW 25.7 | Ampl n.a.  DL n.a.  CV n.a. | Ampl 14.6  DL 3.52  CV 44.7  FW 45.6 |
| **6** | Ampl 13.2  DL 3.37  CV **47.0**  FW **36.9** | Ampl **4.6**  DL **8.74**  CV **38.1** | Ampl 9.7  DL **6.00**  CV **38.9**  FW **64.5** |
| **7** | Ampl 8.8  DL 3.32  CV 49.1  FW 31.0 | Ampl 6.9  DL 4.81  CV 43.5 | Ampl 6.8  DL 4.60  CV 48.8  FW 54.6 |
| **8** | Ampl 9.7  DL 2.69  CV 59.4  FW 27.9 | Ampl **0.55**  DL **7.92**  CV 50.2 | Ampl **5.9**  DL 4.02  CV 51.4  FW n.a. |

Note: for space limits, only distal latencies, distal amplitudes and distal conduction velocities are reported for ulnar and peroneal nerve.

**Table S2:** **Antidromic** **sensory conduction studies.** Ampl=amplitude (µV); DL=distal latency (ms); CV=conduction velocity (ms); n.a.=not applicable. Values abnormal according to our laboratory reference normative values are marked in bold

| **Patient**  **#ID** | **Radial** | **Median** | **Ulnar** | **Suralis**  **(right)** | **Suralis**  **(left)** |
| --- | --- | --- | --- | --- | --- |
| **1** | Ampl 19.6  DL 2.33  CV 51.5 | Ampl 22.8  DL 2.85  CV 49.1 | Ampl **14.2**  DL 2.6  CV 48.1 | Ampl **2.2**  DL 2.62  CV 45.8 | Ampl **4.3**  DL 2.56  CV 54.7 |
| **2** | Ampl 20.1  DL 2.52  CV 49.6 | Ampl 33.3  DL 2.96  CV 47.3 | Ampl 31.9  DL 2.64  CV 43.6 | Ampl 9.4  DL 3.00  CV 40.0 | Ampl 7.1  DL 2.62  CV 53.4 |
| **3** | Ampl 8.9  DL 1.99  CV 55.3 | Ampl 38.3  DL 2.84  CV 47.5 | Ampl 26.0  DL 2.43  CV 43.2 | Ampl 7.4  DL 3.67  CV **38.1*** | Ampl 7.1  DL 3.70  CV **37.8*** |
| **4** | Ampl 16.7  DL 1.94  CV 51.5 | Ampl 39.7  DL 3.15  CV 47.6 | Ampl 27.5  DL 2.58  CV 46.5 | Ampl **6.3**  DL 3.18  CV 40.9 | Ampl n.a.  DL n.a.  CV n.a. |
| **5** | Ampl 16.1  DL 2.08  CV 57.7 | Ampl 44.4  DL 2.25  CV 60.0 | Ampl 50.1  DL 1.97  CV 58.4 | Ampl **4.5**  DL 1.94  CV 56.7 | Ampl n.a.  DL n.a.  CV n.a. |
| **6** | Ampl 17.4  DL 2.92  CV 47.9 | Ampl 51.9  DL 3.13  CV 47.9 | Ampl 18.2  DL 3.00  CV 41.7 | Ampl 8.6  DL 3.08  CV 45.5 | Ampl 11.5  DL 2.36  CV 42.4 |
| **7** | Ampl **7.5**  DL 1.99  CV 50.3 | Ampl 26.1  DL 2.79  CV 50.2 | Ampl **6.6**  DL 2.92  CV 44.5 | Ampl 11.1  DL 2.75  CV 50.9 | Ampl 15.0  DL 2.88  CV 48.6 |
| **8** | Ampl 11.3  DL 2.96  CV 37.2 | Ampl 24.9  DL 3.43  CV 42.3 | Ampl 18.2  DL 2.48  CV 50.4 | Ampl 9.9  DL 3.63  CV **38.6** | Ampl **4.2**  DL 2.25  CV 60.0 |

*sensory nerve conduction velocities are slightly reduced due to cool skin temperature in distal legs (i.e.: 31°C)

**Table S3: EMG rest activity analysis.**

|  | **Tibialis anterior** | | | **Vastus lateralis** | | |
| --- | --- | --- | --- | --- | --- | --- |
| **Patient**  **ID #** | **PSW** | **Fibrillations** | **Fasciculations** | **PSW** | **Fibrillations** | **Fasciculations** |
| **1** | 2/10 | 0/10 | / | 3/10 | 0/10 | / |
| **2** | 0/10 | 0/10 | / | 0/10 | 0/10 | / |
| **3** | 0/10 | 0/10 | / | 0/10 | 0/10 | / |
| **4** | 3/10 | 0/10 | / | 0/10 | 0/10 | / |
| **5** | 4/10 | 4/10 | / | 0/10 | 3/10 | / |
| **6** | 0/10 | 0/10 | / | 0/10 | 0/10 | / |
| **7** | 0/10 | 0/10 | / | 0/10 | 0/10 | / |
| **8** | 2/10 | 0/10 | / | 0/10 | 0/10 | / |

**Table S4: EMG IP analysis.** NSS=number of short segments; values are presented as means when not otherwise indicated**.**

|  | **Tibialis anterior** | | | | | **Vastus lateralis** | | | | |
| --- | --- | --- | --- | --- | --- | --- | --- | --- | --- | --- |
| **Patient**  **ID #** | **Epoch**  **(n°)** | **Turns**  **(mean)** | **Amplitude μV**  **(mean)** | **NSS**  **(mean)** | **Envelope**  **μV (mean)** | **Epoch**  **(n°)** | **Turns**  **(mean)** | **Amplitude μV**  **(mean)** | **NSS**  **(mean)** | **Envelope**  **μV (mean)** |
| **1** | 40 | 370.3 | 299.2 | 296.2 | 914.0 | 33 | 592.0 | 585.2 | 498.3 | 1880.3 |
| **2** | 30 | 437.5 | 838.9 | 278.8 | 3009.5 | 20 | 474.2 | 1244.3 | 332.1 | 4213.8 |
| **3** | 66 | 475.2 | 564.2 | 380.1 | 1843.4 | 38 | 476.3 | 764.2 | 384.4 | 2220.3 |
| **4** | n.a. | n.a. | n.a. | n.a. | n.a. | n.a. | n.a. | n.a. | n.a. | n.a. |
| **5** | n.a. | n.a. | n.a. | n.a. | n.a. | n.a. | n.a. | n.a. | n.a. | n.a. |
| **6** | 35 | 728.9 | 797.0 | 563.5 | 2991.1 | 39 | 568 | 698.3 | 461.0 | 2888.3 |
| **7** | 37 | 519.2 | 658.5 | 355.2 | 2383.3 | 32 | 500.1 | 1094.4 | 335.3 | 3461.2 |
| **8** | 25 | 549.6 | 573.1 | 438.2 | 2116.3 | 29 | 558.5 | 990.0 | 387.4 | 3619.0 |

*for patients #4 and #5 IP analysis was not performed due to insufficient compliance of the patients. In these subjects only a qualitative analysis of rest activity and analysis of motor unit potentials was performed.
